# Ultra-low background radiation and reproduction/development: biological homeostasis, developmental sensitivity, and emerging radiobiological questions

**DOI:** 10.3389/fpubh.2026.1997634

**Published:** 2026-10-22

**Authors:** Shiqi Bian, Yiyao Wu, Xuehong Wan, Hongqian Liu

**Affiliations:** 1 Department of Medical Genetics, West China Second University Hospital, Sichuan University, Chengdu, China; 2 Key Laboratory of Birth Defects and Related Diseases of Women and Children, Sichuan University, Chengdu, China; 3 West China School of Medicine, Sichuan University, Chengdu, China; 4 Deep Underground Space Medical Center, West China Hospital, Sichuan University, Chengdu, China

**Keywords:** developmental biology, DNA damage response, low-dose radiation, oxidative stress, radiation biology, radiation protection, reproductive development, ultra-low background radiation

## Abstract

Natural background radiation has accompanied life since its origin and represents a persistent environmental factor derived from cosmic rays, terrestrial radionuclides, radon, food, and water. While conventional radiobiology has focused primarily on the harmful effects of radiation exposure above background levels, far less attention has been paid to the biological consequences of reducing radiation below naturally occurring terrestrial levels. Recent experiments conducted in deep underground laboratories have enabled investigation of ultra-low background radiation (ULBR), providing a unique opportunity to determine whether natural background radiation is merely an unavoidable environmental exposure or contributes actively to biological homeostasis. Accumulating evidence indicates that ULBR can influence cellular proliferation, reproductive fitness, embryonic development, and adaptive stress responses, although the direction and magnitude of these effects appear strongly dependent on species, developmental stage, exposure duration, and physiological context. Current mechanistic evidence suggests that perturbation of oxidative homeostasis, altered DNA damage response, and mitochondrial metabolic adaptation are the most plausible pathways linking reduced background radiation to biological change. From the perspective of reproductive biology, ULBR is of particular interest because germ cells and early embryos are highly sensitive to subtle disturbances in redox signaling, proliferative control, and genome stability. Available data suggest that the biological consequences of background radiation deprivation may be manifested less through overt toxicity than through subtle shifts in fertility, developmental tempo, and metabolic allocation; the possibility of long-term developmental programming remains an attractive but currently untested hypothesis. This review summarizes current knowledge regarding the biological effects of ULBR, with particular emphasis on reproductive and developmental systems, discusses major mechanistic hypotheses, and highlights critical unresolved questions for future reproductive radiobiology.

## 1 Introduction

Natural background radiation is an omnipresent component of the terrestrial environment and arises primarily from cosmic rays, terrestrial radionuclides, radon gas, food, water, and building materials. According to the United Nations Scientific Committee on the Effects of Atomic Radiation (UNSCEAR), the global average annual effective dose from natural sources is approximately 2.4 mSv, although considerable geographical variation exists depending on altitude, geology, and local environmental conditions (1).

Because life emerged and evolved under continuous exposure to this persistent low-level radiation field, natural background radiation has long been considered a potentially relevant evolutionary factor rather than a biologically negligible environmental constant. Over evolutionary timescales, continuous exposure to low-intensity ionizing radiation may have contributed to the shaping of cellular defense systems, including antioxidant regulation, redox signaling networks, DNA damage surveillance, and adaptive stress responses (2, 3). In this context, natural background radiation may not simply represent an unavoidable environmental exposure, but rather part of the physicochemical conditions under which biological homeostasis evolved. Traditional radiobiology has focused predominantly on the harmful consequences of increased radiation exposure, particularly at moderate and high doses where DNA damage, carcinogenesis, and tissue injury are well documented (4–6). By contrast, considerably less attention has been paid to the opposite question: what are the biological consequences of reducing or nearly eliminating the natural radiation background? This question has given rise to growing interest in ultra-low background radiation (ULBR) research, in which organisms are maintained under radiation environments substantially below normal terrestrial levels (2, 7).

Experimental investigation of ULBR has become possible through the development of deep underground laboratories (DULs), including facilities such as Gran Sasso National Laboratory, SNOLAB, and China Jinping Underground Laboratory. In these facilities, thick layers of rock provide substantial shielding from cosmic radiation, reducing the cosmic-ray component by several orders of magnitude and thereby creating radiation environments far below those encountered at the Earth’s surface (7, 8). Such environments offer a rare opportunity to test whether natural background radiation merely represents an unavoidable physical exposure or instead contributes actively to the maintenance of normal cellular physiology.

In classical low-dose radiation experiments, a defined amount of radiation is typically added above natural background levels; by contrast, ULBR experiments examine the biological consequences of background radiation deprivation itself (2, 9). This distinction is conceptually important, because it reframes the scientific question from how much damage is caused by added radiation to whether the absence of naturally occurring radiation alters biological homeostasis.

Among all biological systems, reproductive tissues and developing embryos are considered particularly sensitive to subtle environmental perturbations, because they depend on precisely coordinated programs of cell proliferation, differentiation, DNA replication, epigenetic remodeling, and metabolic regulation (10–14). Gametogenesis and early embryogenesis are characterized by rapid cell-cycle progression, extensive chromatin reorganization, and tightly regulated mitochondrial activity, processes that are highly dependent on balanced redox signaling and genome maintenance (10, 11, 14–17). Even modest disturbances in intracellular oxidative homeostasis or DNA damage response during these critical windows may lead not only to altered growth kinetics, but also to persistent effects on fertility, developmental programming, and long-term offspring phenotype (10, 13, 18–20).

From a reproductive biology perspective, the study of ULBR is therefore especially compelling. Unlike other somatic cells, reproductive cells and early embryos possess limited tolerance for replication stress, redox imbalance, and mitotic error, making them plausible candidates for detecting subtle biological consequences of background radiation deprivation (10, 11, 18, 21, 22). Emerging evidence from model organisms already suggests that reduced background radiation may influence fertility, egg-laying behavior, embryonic growth, and developmental timing (23–25). These observations raise an important possibility: natural background radiation may contribute to maintaining physiological homeostasis during reproduction and development.

Relevant literature was identified through searches of PubMed, Web of Science, and Google Scholar using terms including ULBR, low background radiation, below-background radiation, and underground laboratory, in combination with reproductive, developmental, and radiobiological terms. Titles and abstracts were screened, and peer-reviewed original studies and authoritative reviews reporting biological endpoints under reduced-background or shielded conditions were included. Additional literature on general radiobiology, reproductive and developmental biology, and the molecular mechanisms discussed in this review was also consulted to provide context and interpret the findings.

In this review, we summarize current evidence regarding the biological effects of ULBR, with particular emphasis on reproductive and developmental systems. We further discuss potential mechanistic pathways, unresolved controversies, and future directions for reproductive radiobiology in ultra-low radiation environments.

## 2 Ultra-low background radiation: experimental definition and context

Ultra-low background radiation differs conceptually from conventional low-dose radiation exposure. In classical low-dose studies, radiation is added above natural background levels, whereas ULBR experiments aim to investigate the biological consequences of reducing the naturally existing radiation background itself (2, 7). In practice, ULBR experiments are typically conducted in deep underground laboratories, where rock overburden substantially attenuates the cosmic-ray component of environmental radiation and thereby creates radiation conditions significantly below those encountered at the Earth’s surface (7, 8, 26). However, underground experimental systems also differ from above-ground laboratories in several environmental parameters, including radon concentration, humidity, ventilation, trace gas composition, and geological microenvironment. These differences complicate causal attribution and remain a major methodological limitation in underground radiobiology (7, 8, 26, 27).

Reported radiation levels varied substantially across experimental systems and underground facilities. In representative deep-underground studies, total or component-specific radiation dose rates were reduced to levels substantially below those measured at surface sites, with the magnitude of reduction depending on the facility and the radiation component assessed. The reported surface and underground exposure levels, measured radiation components, and degree of radiation reduction for individual studies are summarized in Supplementary Table S1. Because several historical studies did not report complete dosimetric information, direct quantitative comparisons between experiments remain limited.

## 3 Effects of ULBR on reproduction and development

### 3.1 Cellular proliferation and growth

Altered cellular proliferation is one of the most frequently investigated and context-dependent biological responses reported under ULBR conditions (28–30). Some of the earliest observations suggesting biological sensitivity to reduced radiation background emerged from underground experiments conducted in the Simplon tunnel, where the cyanobacterium *Mastigocladus laminosus* failed to survive under radiation-shielded conditions (31). Subsequent controlled work by Planel and colleagues prolonged cultivation under radiation-shielded conditions and observed reduced cell multiplication compared with surface controls (32, 33). These early observations provided preliminary evidence that prolonged shielding from environmental radiation may alter cellular survival and proliferation, while also raising the hypothesis that natural background radiation could contribute to the regulation of cellular growth.

Later studies extended these observations to mammalian systems. In a landmark experiment, Kawanishi et al. (28) demonstrated that both *Paramecium* and mouse lymphoma L5178Y cells cultured under reduced background radiation exhibited significant growth retardation after approximately 40–50 days of continuous exposure. Notably, the delayed onset of the phenotype is consistent with a gradual physiological or homeostatic response rather than an immediate cytotoxic effect. More recent experiments have further strengthened this conclusion. In Chinese hamster V79 cells, culture under below-background radiation conditions significantly reduced proliferation rate and altered cell-cycle distribution (29). Parallel work in human laryngeal squamous carcinoma FD-LSC-1 cells showed comparable proliferative suppression accompanied by pronounced ultrastructural remodeling, including mitochondrial enlargement, reduced mitochondrial cristae, and endoplasmic reticulum hypertrophy (30). Latest research on NP69 Human Nasopharyngeal Epithelial Cells and head and neck tumor cells has shifted its focus beyond normal cells, but it has still observed inhibition of proliferation (34).

However, proliferative responses to ULBR are not universally inhibitory. In *Caenorhabditis elegans*, below-background radiation was associated with faster larval development and faster population growth (25). Similarly, lake whitefish embryos were larger when cultured under below-background radiation conditions (24). These apparently divergent outcomes indicate that biological responses to reduced background radiation are heterogeneous and may depend on species, developmental stage, exposure duration, and the balance between stress adaptation and metabolic compensation.

Taken together, current evidence indicates that cellular proliferation is a sensitive but context-dependent endpoint in ULBR research. Rather than producing a uniform suppressive effect, reduction of natural background radiation appears capable of modulating proliferative homeostasis in multiple directions. For reproductive and developmental biology, this is particularly important because proliferation lies at the core of gametogenesis, embryonic cleavage, and tissue morphogenesis. Even subtle shifts in proliferative dynamics under ULBR may therefore have amplified consequences at later developmental stages.

### 3.2 Reproductive fitness and gametogenesis: evidence, knowledge gaps, and biological implications

Although most ULBR studies have focused on general cellular physiology, a smaller but particularly important group of investigations has addressed reproductive fitness, an endpoint that may be more sensitive than somatic growth to subtle environmental perturbation. Reproduction requires precise coordination of gametogenesis, hormonal signaling, meiosis, fertilization competence, and early developmental support. Because these processes depend heavily on redox balance, genome integrity, and tightly regulated cell-cycle progression, they are biologically plausible targets of background radiation deprivation (10, 15–17, 21, 22).

Germ cells are highly sensitive to disturbances in intracellular homeostasis, and alterations in redox balance, genome integrity, or cellular metabolism can affect reproductive competence and subsequent developmental potential. Evidence from conventional radiation biology has demonstrated that germ cells are highly sensitive to radiation exposure. Whether reducing environmental radiation below natural background levels directly influences gametogenesis remains unknown. However, findings from cellular and model-organism studies provide biological plausibility for such an effect. Under reduced-background radiation conditions, cells exhibit altered oxidative stress responses (29, 30, 35, 36). Transcriptomic analyses in *C. elegans* also identified differential expression of genes associated with reproduction, sperm-related functions, stress responses, and metabolic regulation (25).

Among currently available experimental models, the strongest evidence for reproductive sensitivity under ULBR comes from *Drosophila melanogaster*. In the FLYINGLOW project, long-term exposure to reduced natural background radiation was associated with a substantial reduction in fertility in the experimental *Drosophila* populations, with effects persisting across generations (23). This divergence between individual survival and reproductive output is particularly noteworthy. From a life-history perspective, one possible interpretation is that chronic exposure to a low-background environment may alter the balance between somatic maintenance and reproductive investment. Such a trade-off is consistent with broader ecological and evolutionary theories of energy allocation under environmental stress (9, 23).

In contrast, *Caenorhabditis elegans* maintained under below-background radiation conditions showed faster larval development and increased early egg-laying (25). However, no significant difference in age at first reproduction was observed. Transcriptomic analyses at 72 h revealed differential expression of genes associated with development, stress responses, metabolism, and reproductive function.

Whether these findings reflect adaptation, compensation, or altered homeostasis remains unresolved. These observations further indicate that the direction and magnitude of reproductive responses to reduced background radiation may vary substantially across species and experimental contexts.

Despite these informative model-organism studies, direct mechanistic evidence in mammalian reproductive systems remains notably scarce. It remains unclear whether reduced background radiation primarily affects germ-cell proliferation, meiotic chromosome behavior, gamete quality, endocrine regulation, or reproductive tract physiology. Nor is it known whether the fertility alterations observed under ULBR arise from direct effects on the germline or indirectly through broader metabolic and systemic changes (2, 7, 20). Current evidence therefore supports the view that fertility may represent one of the most sensitive biological readouts of ULBR, although the molecular basis of this sensitivity remains largely unresolved.

### 3.3 Early embryonic development and embryo implantation

Embryo implantation and early embryonic development are critical windows during which subtle environmental perturbations can have disproportionately large consequences (37, 38). These stages involve rapid cell division, lineage specification, epigenetic reprogramming, and metabolic transitions that depend on precisely regulated redox homeostasis, DNA replication fidelity, and mitochondrial function (10, 11, 14, 18, 39–42). Because these tightly coordinated processes occur within a narrow developmental timeframe, even subtle perturbations can alter developmental trajectories without producing overt lethality or gross malformation (11, 18–20, 43).

Experimental evidence supporting developmental changes under deep-underground conditions has emerged most clearly from aquatic vertebrate models. In the REPAIR project, embryos of lake whitefish (*Coregonus clupeaformis*) reared in a deep underground environment displayed measurable alterations in developmental phenotype compared with surface controls (24). Specifically, underground-reared embryos were up to 10% greater in body length and mass, and showed reduced yolk mass and area at selected developmental stages (24). No significant difference was observed in survival rate or in time to 50% hatch in the 5 degrees C groups; a reasonable comparison at 3 degrees C was prevented by equipment failure. These developmental differences were associated with underground rearing conditions. However, because radon concentrations and air pressure differed between underground and surface environments, the independent contribution of reduced background radiation remains uncertain.

These findings are biologically important for several reasons. First, reduced yolk mass and area at selected developmental stages suggest altered embryonic energy metabolism, which is consistent with the growing view that ULBR may influence mitochondrial physiology and cellular bioenergetics. Second, the increased embryonic size in the absence of improved survival raises the possibility that underground environmental conditions may influence developmental trajectories or energy allocation beyond simple effects on survival. In other developmental contexts, changes in metabolic partitioning, oxidative signaling, or proliferative timing can influence later organogenesis and tissue differentiation; whether comparable processes occur under ULBR remains to be established.

Current evidence does not permit conclusions regarding implantation success, trophoblast function, or embryo–maternal communication under ULBR conditions. Most existing ULBR studies have focused on cellular proliferation, fertility, or developmental outcomes rather than implantation itself. Consequently, whether reduced background radiation influences implantation success, trophoblast function, or embryo–maternal communication remains unknown. At present, however, the developmental evidence remains limited in both taxonomic breadth and mechanistic depth. Most available studies rely primarily on relatively coarse developmental endpoints such as survival, body size, body mass, and hatching time (7, 24, 25). From the perspective of reproductive and developmental biology, current evidence therefore suggests that ULBR may influence embryonic development primarily through modulation of developmental tempo, metabolic allocation, and early physiological programming rather than overt embryotoxicity. This distinction is crucial. If confirmed, it would imply that the biological consequences of background radiation deprivation may be most meaningfully detected not by gross developmental failure, but by subtle shifts in developmental trajectories that shape later-life phenotype. Although current evidence remains limited, available studies suggest that embryonic developmental processes may be sensitive to alterations in environmental radiation background. Given the central importance of implantation and early embryogenesis for subsequent fetal development, these stages represent important targets for future ULBR research. Further studies in mammalian reproductive models will be required to determine whether background radiation deprivation influences implantation efficiency, developmental competence, or later developmental outcomes.

### 3.4 Developmental trajectories and potential long-term consequences

Compared with cellular and reproductive responses, evidence regarding the effects of ULBR on long-term offspring outcomes remains relatively limited. The available studies suggest that developmental processes may be particularly sensitive to reductions in environmental radiation levels, raising the possibility that ULBR influences not only immediate developmental phenotypes but also longer-term biological trajectories.

Evidence for longer-term developmental consequences has also emerged from studies in the nematode *Caenorhabditis elegans*. In Van Voorhies et al. (25), animals maintained under below-background radiation conditions showed faster larval development and increased early egg-laying compared with worms maintained under normal background radiation. Transcriptomic analyses at 72 h revealed widespread differential expression of genes involved in metabolism, stress responses, development, and reproductive function (25). These findings raise the possibility that reduced background radiation may influence developmental trajectories, although direct evidence for persistent developmental programming under ULBR remains lacking.

The significance of these observations extends beyond the immediate phenotypic changes observed in experimental models. Developmental biology increasingly recognizes that environmental conditions encountered during critical developmental windows can exert lasting influences on physiology, metabolism, reproductive capacity, and disease susceptibility later in life. This concept, commonly referred to as the Developmental Origins of Health and Disease framework, proposes that early-life environmental exposures can shape long-term biological outcomes through developmental programming mechanisms. Although direct evidence linking ULBR to developmental programming in mammals is currently unavailable, the transcriptomic differences measured at 72 h in *C. elegans* are conceptually consistent with this framework, and persistent reprogramming remains a hypothesis to be tested. The transcriptomic differences observed at 72 h represent a time-specific molecular response and do not establish persistent developmental reprogramming or long-term offspring effects.

An additional unresolved question concerns the possibility of multigenerational or transgenerational effects of background radiation deprivation. Evidence from the FLYINGLOW project demonstrated that *Drosophila melanogaster* populations maintained under reduced natural background radiation exhibited alterations in fertility that persisted across successive generations (23). While these observations do not constitute definitive evidence of transgenerational inheritance, they suggest that prolonged exposure to ULBR may influence biological processes that extend beyond a single generation. Potential mechanisms may include persistent epigenetic modifications, altered germ-cell programming, or stable adaptive responses, although these possibilities remain largely unexplored (Figure 1 and Table 1).

**Figure 1:**
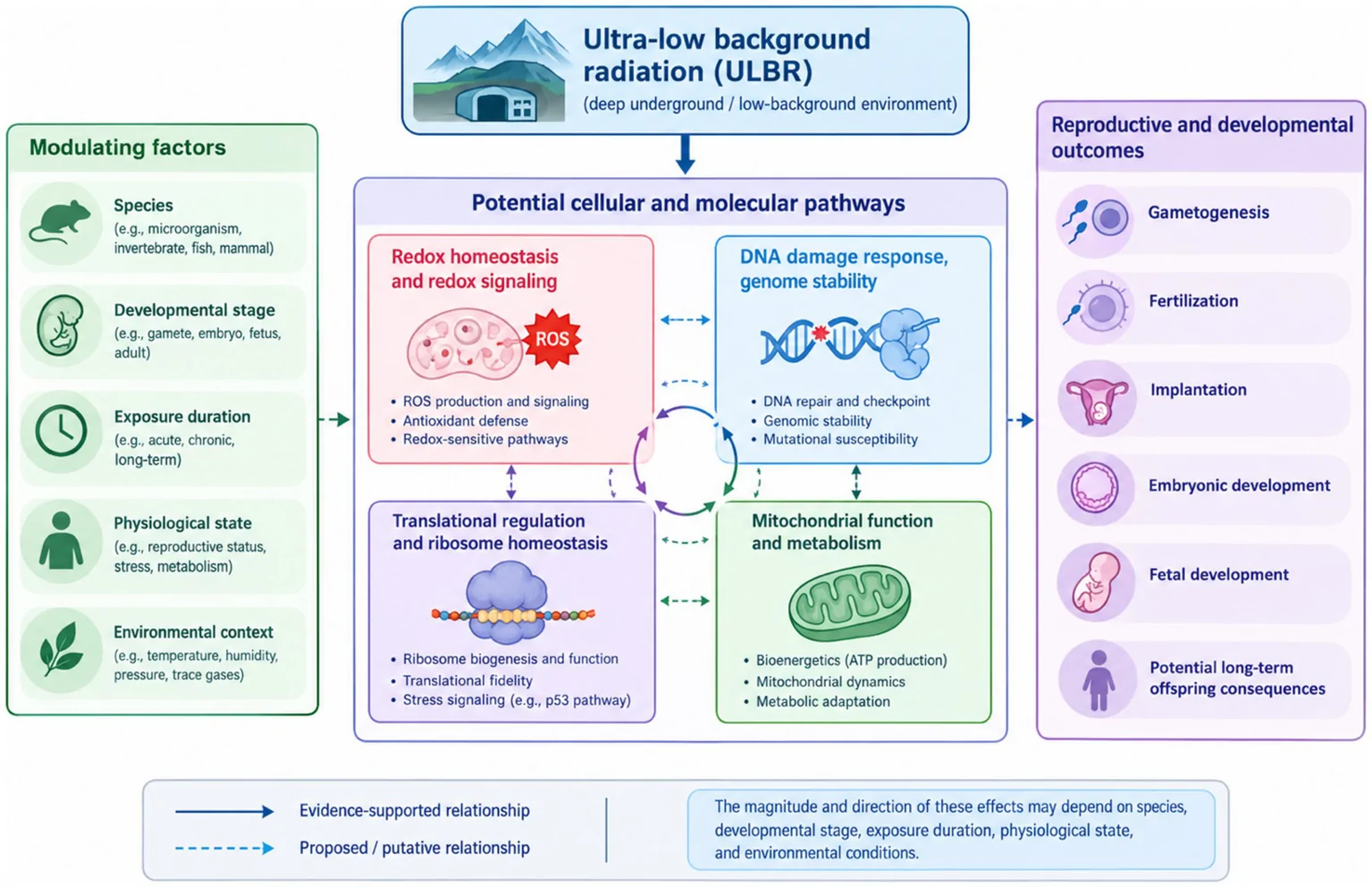
Proposed mechanistic framework linking ultra-low background radiation (ULBR) to reproductive and developmental outcomes. ULBR may influence interconnected cellular pathways involving redox signaling, DNA damage response and genome stability, translational regulation and ribosome homeostasis, and mitochondrial function. These pathways may contribute to changes in gametogenesis, fertilization, implantation, embryonic and fetal development, and potentially long-term offspring phenotypes. The framework integrates direct ULBR observations in reproductive and developmental models where available, observations from other experimental systems, and established general biological mechanisms. Solid arrows indicate relationships directly supported by experimental observations, whereas dashed arrows indicate relationships inferred from other experimental systems or established biological mechanisms and requiring further validation under ULBR conditions. The magnitude and direction of these effects may depend on species, developmental stage, exposure duration, physiological state, and environmental conditions.

**Table 1:** Summary of reported biological responses to ultra-low background radiation (ULBR) across experimental model systems.

Model system and endpoint	Reported finding under ULBR	Biological interpretation	Source	Evidence category
*Mastigocladus laminosus* (cyanobacterium) — Survival under radiation shielding	Failed to survive under conditions shielded from natural background radiation in the Simplon tunnel	Earliest indication that background radiation deprivation can be lethal to simple organisms	Eugster (31)	*A*
Mammalian cell cultures (various) — Cell multiplication rate	Reduced cell multiplication after prolonged culture under radiation-shielded conditions	Early evidence that background radiation may support normal proliferative activity	Planel et al. (32, 33)	*A*
*S. cerevisiae* — DNA adaptive response	Higher recombination after radiomimetic challenge following growth under reduced background radiation	Suggests altered conditioning/adaptive response to genotoxic stress	Satta et al. (46)	*A*
Chinese hamster V79 cells — Mutation frequency, antioxidant capacity, cell density	Increased basal HPRT mutation frequency and sensitivity to mutagenic challenge after 9 months underground; altered catalase/GPx/SOD activity; increased density at confluence	Long-term ULBR exposure is associated with altered genomic stability and antioxidant responses and shifts antioxidant enzyme balance	Satta et al. (36)	*A*
Chinese hamster V79 cells — Mutation frequency persistence; protective mechanisms	Elevated spontaneous HPRT mutation frequency persisted after return to surface conditions	Implicates stable adaptive or suggesting a persistent cellular adaptation that may involve stable regulatory or epigenetic changes rather than transient radiation effect	Fratini et al. (35)	*A*
Human TK6 lymphoblastoid cells — Adaptive response to genotoxic stress	Reduced adaptive response following subsequent genotoxic challenge after culture under reduced background radiation	Suggesting altered maintenance of adaptive stress responses	Carbone et al. (48)	*A*
Mouse lymphoma L5178Y cells; *Paramecium* — Cell growth kinetics	Significant growth retardation after approximately 40–50 days of continuous shielding from background radiation	The delayed onset is consistent with a time-dependent adaptive or homeostatic response rather than an immediate cytotoxic effect.	Kawanishi et al. (28)	*A*
Chinese hamster V79 cells — Proliferation, cell-cycle distribution, ultrastructure	Reduced proliferation rate, altered cell-cycle distribution, mitochondrial enlargement, reduced cristae density, ER dilation; enrichment of oxidoreductase-related proteins	Below-background radiation was associated with altered redox and mitochondrial structure and proteomic profiles; whether these changes reflect impaired bioenergetic function requires direct functional measurements.	Liu et al. (29)	*A*
Human laryngeal squamous carcinoma (FD-LSC-1) cells — Proliferation and ultrastructure	Proliferative suppression with mitochondrial swelling and endoplasmic reticulum hypertrophy	Supports the involvement of mitochondrial and proteomic remodelling in cellular responses observed under deep-underground, low-background conditions	Liu et al. (30)	*A*
*Drosophila* larval neuroblasts (LNGS) — DNA damage response (chromosomal breaks)	Increased susceptibility to radiation-induced chromosomal breaks; rescued by supplemental low-dose γ-irradiation restoring surface-equivalent dose rate	Provides evidence that environmental radiation dose rate can modulate cellular sensitivity to DNA damage	Porrazzo et al. (47)	*A*
*Drosophila melanogaster* (FLYINGLOW) — Fertility, lifespan, development	Reduced fertility in some experimental strains, with effects persisting across generations	Suggests altered reproductive investment under chronic low-background conditions; the underlying physiological basis remains unresolved.	Morciano et al. (23)	*A*
*Caenorhabditis elegans* — Development, reproduction, transcriptome	Faster larval development and increased early egg-laying; transcriptomic differences in metabolic, stress-response, and reproductive genes at 72 h	Indicates altered reproductive and developmental physiology; whether these changes represent adaptation, compensation, or impairment remains unresolved.	Van Voorhies et al. (25)	*A*
Lake whitefish (*Coregonus clupeaformis*) embryos (REPAIR/SNOLAB) — Embryonic growth and development	Up to 10% greater body length and mass; reduced yolk mass and area at selected developmental stages	Deep-underground exposure was associated with altered embryonic growth and reduced yolk mass and area at selected stages; the contribution of reduced radiation versus other underground environmental factors remains unresolved.	Pirkkanen et al. (24)	*B*
NP69 human nasopharyngeal epithelial cells — Proliferation and migration	Inhibition of proliferation and migration in a deep underground low-background environment	Extends proliferative-suppression findings to a normal (non-malignant) human epithelial cell line	Hao et al. (34)	*B*

ULBR, ultra-low background radiation; DDR, DNA damage response; HPRT, hypoxanthine-guanine phosphoribosyltransferase; LNGS, Laboratori Nazionali del Gran Sasso; SOD, superoxide dismutase; GPx, glutathione peroxidase; ER, endoplasmic reticulum. Studies are ordered approximately from earliest/simplest experimental systems to more recent mammalian and human cell models. For exposure duration, comparison parameters, radiation measurements, and major limitations of the principal studies, see Supplementary Table S1. Full bibliographic details are provided in the reference list. Evidence categories: A, direct experimental evidence obtained under reduced-background or radiation-shielded conditions; B, deep-underground or low-background evidence for which attribution to radiation deprivation is limited by environmental confounding. These categories describe the strength of attribution of the reported effect to radiation deprivation, not overall study quality. Studies classified as A or B demonstrate biological responses under low-background conditions but do not necessarily establish that radiation deprivation is the sole causal factor; even category A studies may have residual environmental confounding.

### 3.5 Oxidative homeostasis and redox signaling

Among the mechanistic hypotheses proposed to explain the biological effects of ULBR, perturbation of oxidative homeostasis is currently one of the most plausible and widely discussed (2, 3, 15–17, 27). Traditionally, ionizing radiation has been viewed primarily as a source of oxidative damage because of its ability to generate reactive oxygen species (ROS). Contemporary redox biology has established that ROS are not merely toxic by-products of metabolism. At physiological concentrations, they function as essential signaling mediators that regulate cell proliferation, differentiation, mitochondrial communication, DNA repair, apoptosis, and adaptive stress responses (3, 15–17, 44).

Under normal terrestrial conditions, living organisms are continuously exposed to very low levels of ionizing radiation. Although the absolute contribution of background radiation to total intracellular ROS production is small relative to endogenous mitochondrial metabolism, chronic low-level irradiation has nevertheless been proposed to participate in maintaining a basal redox signaling tone (2, 16, 44, 45). From this perspective, the biological consequence of ULBR may not simply be a reduction in oxidative injury, but rather a disturbance of the signaling equilibrium that supports cellular homeostasis.

Experimental findings are broadly consistent with this interpretation. In underground biological systems, altered expression of genes involved in oxidative stress regulation, antioxidant defense, and stress-response signaling has been repeatedly observed (29, 30, 35, 36). Proteomic analyses in V79 cells indicated enrichment of proteins involved in oxidoreductase activity and redox processes (29). Mammalian cells cultured under ULBR display ultrastructural mitochondrial changes, including mitochondrial enlargement and reduced cristae density; these changes are compatible with altered redox metabolism, but direct evidence of impaired bioenergetic function is limited (29, 30).

The relevance of redox perturbation is particularly strong in reproductive and developmental biology. Gametogenesis, fertilization, and early embryogenesis all rely on finely balanced ROS signaling (10, 18, 21, 22). In these systems, physiological ROS regulate meiotic progression, sperm capacitation, oocyte maturation, cleavage dynamics, and early lineage specification (10, 14, 18, 21, 22). Both excessive ROS and insufficient ROS signaling can disrupt developmental homeostasis (10, 15–18, 21, 22). Therefore, if ULBR alters basal oxidative signaling sufficiently to modify redox-sensitive cellular processes, it could plausibly influence reproductive competence or early embryonic development, even in the absence of overt oxidative damage.

Oxidative homeostasis should not be interpreted as an isolated mechanism. Redox signaling is tightly integrated with mitochondrial function, cell-cycle regulation, DNA damage surveillance, and epigenetic control (13, 15–17, 44). Consequently, disturbance of oxidative equilibrium under ULBR may represent an upstream event capable of propagating through multiple downstream biological pathways. This integrative role makes redox signaling a particularly useful conceptual framework for understanding why ULBR can produce subtle yet measurable effects across diverse biological systems.

### 3.6 DNA damage response, genome stability, and translational homeostasis

Maintenance of genome integrity is fundamental to cellular survival, proliferative fidelity, and normal reproductive development. Under natural surface conditions, chronic low-level ionizing radiation has been proposed to contribute to a constitutive state of DNA damage response (DDR) readiness, sustaining basal activation of repair pathways, checkpoint surveillance, and adaptive stress responses (2, 44). Ultra-low background radiation, by reducing this environmental stimulus, could alter the maintenance or inducibility of cellular stress-response and DNA repair systems.

Experimental evidence indicates that reduced background radiation can modify cellular responses to genotoxic stress and genome-maintenance phenotypes, although whether these changes reflect attenuation of constitutive DDR activity remains unresolved. In *Saccharomyces cerevisiae*, growth under reduced background radiation increased the frequency of recombination induced by a subsequent radiomimetic challenge, suggesting that reduced background radiation can modify cellular responses to subsequent genotoxic stress (46). More direct mechanistic evidence was obtained from *Drosophila melanogaster* neuroblasts maintained in the LNGS underground laboratory, where cells exhibited increased susceptibility to radiation-induced chromosomal breaks compared with surface controls; notably, this dose-rate dependence indicates that environmental radiation conditions can modulate cellular susceptibility to subsequent DNA damage, although the existence of a minimum radiation requirement for normal DDR function has not been established (47). Similarly, Chinese hamster V79 cells maintained underground for prolonged periods displayed elevated spontaneous HPRT mutation frequencies and increased sensitivity to mutagenic challenge, effects that became progressively apparent during long-term culture (36). Subsequent studies further demonstrated persistence of this altered mutational phenotype even after return to surface conditions, implicating stable adaptive or epigenetic alterations rather than transient radiation effects alone (35). In human TK6 lymphoblastoid cells, reduced background radiation also impaired adaptive responses to subsequent genotoxic stress, further supporting the concept that reduced background radiation can modify constitutive DDR activity (48).

Chronic low-level environmental radiation has been proposed to contribute to the maintenance of repair-pathway activity, antioxidant regulation, and checkpoint sensitivity through continuous low-grade stimulation (2, 45). Whether reduced background radiation alters these processes sufficiently to affect repair efficiency or genome stability over time remains to be established. Importantly, the biological consequences of altered genome maintenance may extend beyond DNA damage itself to broader disturbances in translational regulation and proteostasis.

One potentially relevant downstream pathway is translational homeostasis. Perturbations in genome maintenance, RNA processing, ribosome biogenesis, or translational regulation can alter ribosome composition and translational fidelity. Ribosomal proteins are essential components of ribosome assembly and function, and changes in their abundance or stoichiometry can selectively reshape translational programs rather than simply reducing global protein synthesis (49, 50). Evidence from ribosomopathies and cancer biology further indicates that perturbation of ribosomal composition or biogenesis can influence the translation of mRNAs involved in proliferation, differentiation, and cellular stress responses (51, 52, 65).

Defective ribosome biogenesis can also activate nucleolar stress signaling. In established mammalian systems, the RPL5–RPL11–5S rRNA complex can interact with MDM2 and promote p53 stabilization, thereby linking ribosome biogenesis to cell-cycle arrest and apoptosis (53, 54). These mechanisms provide biological plausibility for a connection between altered cellular homeostasis and translational regulation under ULBR. However, direct evidence that ULBR induces mutations in ribosomal protein genes or activates the RPL5/RPL11–5S–MDM2–p53 pathway is currently lacking. Early embryogenesis and germ-cell development are highly dependent on tightly coordinated translational programs. Prior to activation of the embryonic genome, cleavage-stage embryos rely predominantly on selective translation of maternally deposited mRNAs to regulate developmental progression and lineage specification (10, 14). Similarly, gametogenesis requires accurate coordination of proliferation, meiosis, and differentiation across large numbers of rapidly dividing precursor cells (11). Consequently, even subtle disturbances in translational fidelity, proteostasis, or p53-dependent surveillance pathways may disproportionately influence developmental competence and reproductive fitness.

These findings support the possibility that ULBR may modify cellular stress responses, genome-maintenance processes, and translational homeostasis. However, whether these changes reflect a persistent weakening of constitutive DDR readiness or lead to cumulative genomic instability remains to be established. Although many mechanistic links remain hypothetical and direct evidence in mammalian reproductive systems is still limited, these processes provide plausible mechanistic connections between background radiation deprivation and the altered proliferation, fertility, and developmental phenotypes observed across experimental model systems.

### 3.7 Mitochondrial function and metabolic adaptation

Mitochondria occupy a central position at the intersection of cellular metabolism, redox regulation, proliferation, and developmental signaling. Because they are the principal source of endogenous ROS, the main site of oxidative phosphorylation, and important regulators of apoptosis and calcium homeostasis, mitochondria are especially plausible mediators of biological responses to ULBR (16, 17). Increasing evidence suggests that one consequence of background radiation deprivation may be metabolic adaptation, with mitochondria acting as major cellular sensors and effectors of this process (27, 29, 30). Direct observations from mammalian cells cultured under below-background radiation conditions include mitochondrial enlargement, reduced mitochondrial cristae density, and dilation of the endoplasmic reticulum in Chinese hamster V79 cells (29), and mitochondrial swelling and endoplasmic reticulum hypertrophy in human laryngeal squamous carcinoma FD-LSC-1 cells (30). These structural and proteomic changes are compatible with altered cellular bioenergetics, but they do not by themselves demonstrate impaired bioenergetic function; direct measurements of respiration and ATP production are still required.

The relevance of mitochondrial adaptation is particularly strong in reproductive biology. Gametes and preimplantation embryos rely heavily on tightly regulated mitochondrial function. Oocyte maturation, sperm motility, fertilization competence, cleavage-stage progression, and blastocyst formation all depend on precise control of ATP generation, mitochondrial dynamics, calcium signaling, and ROS-mediated communication (10, 21, 22). Disturbance of mitochondrial homeostasis during these stages can alter not only immediate developmental progression but also long-term developmental programming and offspring phenotype.

Importantly, mitochondrial adaptation under ULBR is unlikely to represent an isolated downstream consequence. Mitochondria are functionally integrated with redox signaling, DNA damage response, and cell-cycle regulation, allowing relatively small perturbations in bioenergetic homeostasis to propagate broadly across cellular physiology (12, 15–17, 44). From this perspective, mitochondrial function may represent one of the principal nodes through which reduced background radiation is translated into measurable biological effects.

Current evidence suggests that metabolic adaptation, particularly at the mitochondrial level, may constitute a major mechanistic link between ULBR and altered proliferative, reproductive, and developmental outcomes. However, direct functional evidence remains limited. Future studies need to determine whether ULBR primarily alters cellular metabolism or whether mitochondrial remodeling reflects secondary adaptation to broader changes in intracellular homeostasis.

## 4 Why are biological responses to ULBR sometimes contradictory?

One of the most striking features of the ULBR literature is the coexistence of apparently contradictory biological outcomes. In some experimental systems, reduced background radiation is associated with suppressed proliferation, impaired adaptive stress response, and reduced fertility (23, 25, 28–30, 35, 48). In others, ULBR has been linked to accelerated growth, increased reproductive output, or enhanced developmental progression (24, 25). Although results like the latter are in the minority, at first glance, these findings may appear mutually incompatible. However, when viewed within the broader context of cellular adaptation and environmental physiology, such heterogeneity is not unexpected.

### 4.1 Species-specific biological sensitivity

Different organisms likely possess fundamentally different thresholds for sensing and responding to altered radiation environments. Basal metabolic rate, endogenous ROS production, genome maintenance strategy, life-history characteristics, and reproductive investment differ markedly across taxa. Consequently, the same reduction in radiation background may not represent the same biological perturbation for protozoa, invertebrates, vertebrate embryos, or mammalian somatic cells. For example, *Drosophila melanogaster* showed reduced fertility under ULBR (23), whereas *Caenorhabditis elegans* exhibited faster larval development and increased early egg-laying (25). These divergent responses may reflect genuine differences in developmental plasticity, stress adaptation capacity, or reproductive allocation rather than experimental inconsistency.

### 4.2 Exposure duration and adaptive timescale

The duration of exposure appears to be another critical determinant of biological outcome. Several studies indicate that measurable effects often emerge only after prolonged exposure, rather than immediately after transfer to a reduced-radiation environment (28, 35, 48). This delayed response suggests that ULBR may not act as an acute damaging stimulus but rather as a chronic environmental condition requiring physiological adaptation. Short-term exposure may reveal little phenotypic change, whereas long-term exposure may progressively reshape metabolic state, proliferative behavior, and stress-response capacity. Under this interpretation, apparently inconsistent results across studies may partly reflect differences in the temporal window of observation.

### 4.3 Developmental stage and endpoint selection

Biological sensitivity to ULBR likely depends strongly on developmental stage. Rapidly proliferating cells, germ cells, early embryos, and differentiating tissues may be substantially more sensitive than terminally differentiated somatic cells because they rely heavily on precise coordination of DNA replication, mitochondrial function, and redox signaling (10, 11, 18, 21, 22).

In addition, different studies often examine distinct biological endpoints, including proliferation rate, fertility, lifespan, mutation frequency, embryo size, hatching time, and resistance to secondary stress. Because these endpoints reflect different levels of biological organization, heterogeneous outcomes should not necessarily be interpreted as contradictory. A reduction in fertility, for example, can coexist with preserved survival or even increased body growth (23, 24).

### 4.4 Underground environmental confounding factors

Perhaps the most important experimental limitation is that underground environments differ from surface laboratories in both radiological exposure and non-radiation variables. Radiological exposure includes not only the reduced gamma-ray and cosmic-ray fields but also radon and its short-lived progeny, which contribute to alpha-particle dose (7, 8). Non-radiation variables include humidity, atmospheric pressure, trace gas composition, ventilation, microbiological environment, and geochemical background (7, 8, 27). These non-radiation variables can independently influence oxidative stress, metabolism, cellular growth, and cellular physiology (7, 27, 55). Accordingly, findings obtained in deep-underground environments should generally be interpreted as evidence of biological responses to low-background underground conditions rather than as definitive evidence attributable exclusively to radiation deprivation, unless relevant environmental variables have been adequately controlled.

## 5 Current controversies and unresolved questions in ULBR biology

### 5.1 Is background radiation biologically necessary?

One of the most fundamental controversies in ULBR research concerns whether natural background radiation is merely an unavoidable environmental exposure or a biologically relevant component of normal physiological homeostasis. The Linear No-Threshold (LNT) model is a radiation-protection model that extrapolates radiation-related stochastic cancer risk linearly to low doses; it does not by itself address whether low-level radiation has physiological benefit (56, 64). Under this risk framework, natural background radiation is viewed solely as a source of stochastic DNA damage rather than a biologically required environmental factor. Observations of altered proliferation, egg-laying, or embryo size under ULBR are biological endpoints and do not directly confirm or disprove the cancer dose–response relationship.

In contrast, accumulating evidence from underground laboratory studies has led some investigators to propose that organisms may have evolved under persistent exposure to natural radiation and therefore utilize low levels of ionizing radiation as part of normal environmental signaling. Experimental observations demonstrating altered proliferation, impaired adaptive responses, increased mutation frequencies, and changes in reproductive performance under ULBR conditions suggest that background radiation may contribute to the maintenance of cellular stress-response systems, redox homeostasis, and DNA repair readiness (2, 44, 57). Whether natural background radiation represents a biologically necessary environmental stimulus or merely a tolerated source of damage remains unresolved and continues to represent one of the central questions in modern radiobiology.

### 5.2 Adaptive response or biological impairment?

The interpretation of biological changes observed under ULBR conditions remains a central unresolved issue. Many studies report measurable alterations in growth, reproduction, and development without evidence of overt toxicity, increased mortality, or gross developmental abnormalities.

Lake whitefish embryos maintained in a deep underground environment exhibited increased body size and reduced yolk mass and area at selected stages despite unchanged survival rates (24). Similarly, *Caenorhabditis elegans* exposed to below-background radiation conditions showed faster larval development and increased early egg-laying, together with transcriptomic differences at 72 h affecting developmental, metabolic, and stress-response pathways (25). In *Drosophila melanogaster*, reduced background radiation altered fertility and developmental characteristics across generations (23).

The biological significance of these observations remains uncertain. One interpretation is that these changes reflect impaired homeostasis caused by deprivation of a normal environmental factor. Alternatively, they may represent adaptive physiological adjustments to a novel environment. Because relatively few studies have examined lifetime fitness, reproductive success, or long-term health outcomes, it remains difficult to determine whether ULBR-induced phenotypes should be considered harmful, beneficial, or simply adaptive. Resolving this issue will be critical for understanding the biological significance of background radiation. Importantly, a measurable biological change should not automatically be interpreted as either beneficial or detrimental; its biological significance depends on whether the change ultimately alters survival, reproductive success, developmental competence, or long-term organismal fitness.

### 5.3 The challenge of extrapolating to human reproductive biology

Although evidence supporting biological responses to ULBR is steadily accumulating, most available data originate from microorganisms, invertebrates, fish, and cultured cells. Direct investigations of mammalian reproduction and development under below-background radiation conditions remain extremely limited. This limitation is particularly important because reproductive and developmental processes—including gametogenesis, fertilization, implantation, placental development, fetal growth, and developmental programming—involve complex interactions among multiple tissues and regulatory pathways. Such processes cannot be fully recapitulated in simple experimental systems. Consequently, the extent to which observations from yeast, nematodes, insects, or fish can be extrapolated to mammals remains uncertain.

At present, there is insufficient evidence to determine whether ULBR has measurable consequences for human reproductive health. Bridging this translational gap represents one of the most important challenges facing the field and should be considered a priority for future research (58).

## 6 Future directions and perspectives

Future progress in ULBR research will depend heavily on the establishment of dedicated mammalian models designed specifically to investigate reproductive and developmental outcomes. Particular attention should be directed toward spermatogenesis, oogenesis, fertilization, embryo implantation, placental development, fetal growth, and postnatal health. Long-term studies using mice and other mammalian systems will be essential for determining whether the developmental alterations observed in lower organisms are conserved in more complex biological systems. The integration of underground research facilities with modern reproductive biology platforms, including organoid cultures and assisted reproduction technologies, may provide particularly valuable experimental opportunities.

Emerging evidence from fish, insects, and nematodes suggests that ULBR may alter developmental trajectories, reproductive schedules, and life-history characteristics. These findings raise the possibility that reduced background radiation influences developmental programming processes. The Developmental Origins of Health and Disease (DOHaD) framework proposes that environmental conditions encountered during critical developmental windows can exert long-lasting effects on adult physiology, metabolism, reproduction, and disease susceptibility (59–61). Although direct evidence linking ULBR to developmental programming is currently lacking, future studies should investigate whether exposure during embryonic and fetal development influences postnatal growth, reproductive fitness, ageing trajectories, or susceptibility to disease. Particular emphasis should be placed on the potential role of epigenetic regulation, including DNA methylation, histone modification, and non-coding RNAs, as mechanisms through which ULBR-induced biological effects might persist across the lifespan or potentially across generations.

Most existing ULBR studies have focused on isolated molecular endpoints or individual physiological traits. Future investigations should increasingly employ systems-level approaches that integrate transcriptomics, epigenomics, proteomics, metabolomics, and single-cell analyses. Such multi-omics strategies have the potential to identify coordinated regulatory networks underlying responses to reduced background radiation and may reveal previously unrecognized links among redox homeostasis, DNA damage responses, translational regulation, cellular metabolism, and developmental processes (62, 63). These approaches may also facilitate the identification of molecular biomarkers that could be used to monitor adaptation to ULBR environments.

Perhaps the most important unanswered question in ULBR biology is whether a biological threshold exists below which further reductions in environmental radiation become detrimental rather than beneficial. Current experimental findings suggest that certain biological processes may be altered when organisms are maintained in environments with radiation levels substantially below those encountered on the Earth’s surface. However, the existence, location, and biological significance of such thresholds remain unknown. Future experiments employing precisely controlled radiation gradients will be required to determine whether an optimal range of background radiation exists for maintaining cellular and organismal homeostasis. Addressing this question would have important implications not only for radiobiology and evolutionary biology but also for radiation protection policy and long-duration human habitation in highly shielded environments, including deep underground facilities and future space habitats.

## 7 Conclusion

Ultra-low background radiation research has opened a distinct dimension of radiobiology by shifting attention from the biological consequences of radiation excess to the potential consequences of reducing environmental background radiation. Evidence from microorganisms, invertebrates, mammalian cell systems, and vertebrate embryos indicates that exposure to below-background radiation conditions can be associated with measurable changes in cellular proliferation, reproductive performance, developmental progression, and adaptive stress responses. However, these findings are heterogeneous across biological systems, and direct attribution to radiation deprivation remains difficult in many deep-underground experiments because of environmental confounding.

Current evidence does not support a uniform biological response to ULBR. Instead, the magnitude and direction of observed effects appear to depend on species, developmental stage, exposure duration, physiological state, endpoint selection, and environmental context. Proposed mechanisms involving redox signaling, DNA damage responses, translational homeostasis, and mitochondrial function provide biologically plausible frameworks for interpreting these observations, but direct mechanistic evidence in reproductive systems remains limited.

From the perspective of reproductive and developmental biology, the major significance of ULBR research may therefore lie not in demonstrating overt developmental toxicity, but in identifying whether sustained reduction of environmental radiation can subtly modify reproductive physiology or developmental trajectories. At present, evidence for direct effects on mammalian gametogenesis, embryo implantation, fetal development, or long-term offspring health remains insufficient. Future studies using rigorously controlled radiation gradients and dedicated mammalian reproductive models will be essential to determine whether the biological responses observed in lower organisms and cultured cells are conserved in mammals and whether they have meaningful consequences for human reproductive health.
