# Supplementary material for: Ultra-low background radiation and reproduction/development: biological homeostasis, developmental sensitivity, and emerging radiobiological questions
Source: Front Public Health. 2026 Oct 22;14:1997634. doi: 10.3389/fpubh.2026.1997634 (PMC13735102; doi:10.3389/fpubh.2026.1997634)
Supplement: Supplementary file 1 [file Table_1.docx]

# 1 Supplementary Material

Supplementary Table S1. Detailed characteristics of principal studies investigating ultra-low background radiation

| **Study / model** | **Exposure duration** | **Comparison parameters** | **Radiation measurement / reduction** | **Major limitations** |
| --- | --- | --- | --- | --- |
| *Mastigocladus laminosus* (Eugster, 1964) | Not specified | Radiation-shielded vs surface conditions | Simplon tunnel (Switzerland), shielded from cosmic radiation; quantitative dose rates not reported | Limited dosimetry; historical replication |
| Mammalian cell cultures (Planel et al., 1976, 1987) | Prolonged culture | Shielded vs surface controls | Radiation-shielded chambers vs surface controls; background components reduced; detailed quantitative dosimetry limited | Variable cell systems; historical environmental control |
| *Saccharomyces cerevisiae* (Satta et al., 1995) | Not specified | Reduced background plus radiomimetic challenge vs normal background | Underground Gran Sasso National Laboratory (LNGS) vs standard surface background; reduced environmental background radiation | Single microbial model |
| Chinese hamster V79 cells (Satta et al., 2002) | Approximately 9 months | Underground vs surface | LNGS underground vs ISS surface: γ-ray exposure reduced ~70-fold (cumulative ~30 µGy vs ~2 mGy over 9 months); radon reduced ~25-fold (~5 vs ~120 Bq/m³) | Long-term cell adaptation; residual environmental confounding |
| Chinese hamster V79 cells (Fratini et al., 2015) | 10 months underground, then 6 months at surface | Underground then surface; mutation frequency compared before/after return | LNGS low-radiation environment (LRE) vs reference-radiation environment (RRE): neutrons reduced ~10³-fold, directly ionizing cosmic rays ~10⁷-fold, total γ-rays ~10-fold | Attribution of persistence to radiation vs other adaptive changes |
| Human TK6 lymphoblastoid cells (Carbone et al., 2009) | 6 months | Low-background laboratory vs surface, with subsequent genotoxic challenge | LNGS underground laboratory (Gran Sasso); cells maintained 6 months at reduced environmental dose rate | No reproductive endpoints |
| *Paramecium* and mouse lymphoma L5178Y cells (Kawanishi et al., 2012) | 40-50 days | Continuous shielding vs surface controls | Shielding chamber (15 cm iron + 10 cm paraffin walls); γ-rays reduced to ~2% and neutrons to ~25% of background | Limited mechanistic characterization; non-reproductive endpoints |
| Chinese hamster V79 cells (Liu et al., 2020a) | Short-term culture (proliferation over 7 days; TEM/proteomics at 2 days) | Underground vs surface; tandem mass tag proteomics | China Jinping Underground Laboratory (DUGL): γ-ray dose rate 0.04 µSv/h (0.035–0.045) vs 0.15 µSv/h (0.13–0.18) above ground; radon 1.5 pCi/L | Cell-line model; no direct mitochondrial functional assays |
| Human laryngeal squamous carcinoma FD-LSC-1 cells (Liu et al., 2020b) | Short-term culture (proliferation over 7 days; TEM at 2 days; proteomics at 4 days) | Underground vs surface | Deep-underground laboratory (CJPL): terrestrial γ-ray dose rate 0.04 µSv/h vs 0.15 µSv/h above ground; cosmic radiation negligible | Tumor cell line |
| *Drosophila* larval neuroblasts (Porrazzo et al., 2022) | Larval development | LNGS vs surface, with supplemental gamma irradiation | LNGS underground (LRE) ~20 nGy/h vs external reference (RRE) ~66 nGy/h (γ ~20 vs ~22 nGy/h; cosmic muons negligible vs ~41 nGy/h) | Neuroblast DNA damage only |
| *Drosophila melanogaster* FLYINGLOW populations (Morciano et al., 2018) | Multigenerational | Underground vs reference populations | LNGS underground vs external reference populations; reduced environmental radiation background (multigenerational) | Population variability; environmental confounding |
| *Caenorhabditis elegans* (Van Voorhies et al., 2020) | Up to 8 months (development to reproductive period) | Background vs below-background radiation | WIPP underground laboratory (New Mexico): below-background incubator ~15.6 nGy/h vs KCl-supplemented ~67.4 nGy/h | Transcriptomics limited to 72-h worms; no age-at-first-reproduction difference |
| Lake whitefish embryos (Pirkkanen et al., 2020) | Embryogenesis (fertilization to hatch) | Underground (REPAIR/SNOLAB) vs surface | SNOLAB, 2 km underground, shielded from cosmic radiation; specific dose rates not reported; radon not controlled | High radon and uncontrolled air pressure; attribution uncertain |
| NP69 human nasopharyngeal epithelial cells (Hao et al., 2025) | Short-term parallel culture (proliferation at 72–96 h; wound healing 36 h) | Deep-underground vs surface | China in situ Deep-Underground Facility & Life Observatory (DeUFO) at 0 m, 1000 m, and 1500 m depth; low-background radiation environment | Normal epithelial cell line; residual environmental confounding |

Notes: LNGS, Laboratori Nazionali del Gran Sasso; ISS, Istituto Superiore di Sanità; SNOLAB, Sudbury Neutrino Observatory Laboratory; WIPP, Waste Isolation Pilot Plant; CJPL, China Jinping Underground Laboratory; DeUFO, China in situ Deep-Underground Facility & Life Observatory; DUGL/AGL, deep-underground/above-ground laboratory; LRE/RRE, low-radiation/reference-radiation environment.
